# Feasibility of a Mobile-Based Home Monitoring System for Patients With Heart Failure: Mixed Methods Pilot Study

**DOI:** 10.2196/84309

**Published:** 2026-07-29

**Authors:** Finna E Indriany, Kemal N Siregar, Bambang Budi Siswanto, Budhi Setianto Purwowiyoto, Rarsari Soerarso, Indrajani Sutedja, Benny Setiawan, Hendy R Wijaya, Raymond Ching Chiew Wong, Salvatore Di Somma, Peter MacDonald

**Affiliations:** 1 Faculty of Public Health, Universitas Indonesia, Jalan Lingkar Kampus UI, Depok, 16424, Indonesia; 2 Cardiology and Vascular Medicine Department, Faculty of Medicine, Universitas Indonesia, Jakarta, Indonesia; 3 Information Systems Department, School of Information Systems, Bina Nusantara University, Jakarta, Indonesia; 4 Pharmacy Study Program, Faculty of Health Sciences, Pelita Harapan University, Tangerang, Indonesia; 5 Research and Technology Transfer Office, Bina Nusantara University, Jakarta, Indonesia; 6 Department of Cardiology, National University Heart Center, Singapore, Singapore; 7 Great Health Science, AISC Italian Association of Patients with Heart Failure, Rome, Italy; 8 St Vincent’s Clinical School, Faculty of Medicine, University of New South Wales, Sydney, Australia

**Keywords:** heart failure, digital health, remote monitoring, Kansas City Cardiomyopathy Questionnaire, pilot study

## Abstract

**Background:**

The risk of rehospitalization in patients with heart failure (HF) has initiated various efforts to prevent and simultaneously improve quality of life. Self-monitoring at home is one option, and technology is increasingly being used for this purpose.

**Objective:**

This pilot study aimed to evaluate the feasibility and preliminary effects of a digital home monitoring intervention on patient-reported outcomes and 30-day readmissions among patients with HF in Indonesia.

**Methods:**

A mixed methods pilot study was conducted, combining qualitative system development and quantitative evaluation. Patients were assigned to an intervention group (digital monitoring) or control group (standard care). Readmission rates were compared using chi-square tests and odds ratios. Changes in Kansas City Cardiomyopathy Questionnaire scores were analyzed using linear mixed-effects models.

**Results:**

A total of 60 patients were included (n=30, 50% in the intervention group; n=30, 50% in the control group). Readmission occurred in 20% (6/30) of patients in the intervention group and 43.3% (13/30) of patients in the control group (odds ratio 0.33, 95% CI 0.10‐1.09; *P*=.10). Linear mixed-effects analysis showed greater improvement in Kansas City Cardiomyopathy Questionnaire overall summary score in the intervention group (*P*=.02). Improvements were observed in the physical limitation, symptom frequency, symptom burden, quality of life, and social limitation domains. During follow-up, 3.3% (1/30) of the patients in the intervention group died of non–HF-related causes, and 10% (3/30) of the patients in the control group died due to HF.

**Conclusions:**

This pilot study suggests that digital home monitoring is feasible and associated with improvements in patient-reported outcomes, with a potential signal toward reduced readmission. Larger studies are needed to confirm effectiveness.

## Introduction

Heart failure (HF) is associated with high morbidity, mortality, and frequent hospital readmissions [1]. It affects over 20 million people worldwide, with a 5-year mortality rate of 60% to 70% [2]. It is a major public health problem, particularly among older adults, with a prevalence of 1% to 2% in low-income countries [3]. Reducing readmissions remains a key priority in HF management [4,5]. Digital health interventions, including home monitoring systems, have been increasingly used to support postdischarge management [6,7]. These systems enable early detection of symptom deterioration and may improve patient self-management. However, most evidence comes from high-income settings, with limited data from resource-limited environments such as Indonesia [8,9]. This study aimed to evaluate the feasibility of a digital home monitoring intervention and explore preliminary effects on patient-reported outcomes and readmissions in a real-world Indonesian setting.

In many health care settings, postdischarge monitoring of patients with HF still relies on periodic outpatient visits and manual patient reporting [10]. This approach limits the ability of health care providers to detect early clinical deterioration. Consequently, innovative strategies are needed to support continuous monitoring and improve patient engagement in self-management [11,12].

Digital health technologies, particularly mobile health apps, have emerged as promising tools to support chronic disease management. Mobile-based monitoring systems enable patients to record symptoms, physiological parameters, and medication adherence while allowing health care providers to review patient-reported data remotely. Several studies have suggested that remote monitoring may improve patient engagement, facilitate early detection of clinical deterioration, and potentially reduce hospital readmissions [13]. However, the implementation of digital monitoring systems in real-world clinical settings remains challenging, particularly in terms of patient adherence, usability, and integration into existing health care workflows.

In Indonesia, the use of mobile-based monitoring apps for HF management is still limited. Understanding feasibility and patient adherence to such systems is essential before larger-scale implementation can be considered [14]. Therefore, this study developed a mobile-based monitoring app called FineHeart to support home monitoring and communication between patients with HF and health care providers.

This study aimed to evaluate the feasibility, patient adherence, and preliminary clinical outcomes associated with the use of the FineHeart mobile app among patients with HF following hospital discharge.

## Methods

### Study Design

This was a mixed methods pilot study consisting of qualitative needs assessment and system development combined with quantitative evaluation of feasibility and preliminary outcomes. This study was conducted from January 1, 2024, to November 30, 2024, at the National Cardiovascular Center (NCVC) Harapan Kita, Jakarta, Indonesia.

### Participants and Setting

A qualitative needs assessment was conducted through interviews with hospital staff and document studies at the NCVC Harapan Kita. For the quantitative study, patients diagnosed with HF were recruited from a clinical setting. Eligibility criteria were applied consistently across groups. Participants were assigned to intervention or control groups based on predefined operational criteria (nonrandomized allocation). Baseline differences between groups were assessed and are acknowledged as potential confounders.

### Intervention

The digital home monitoring system, named FineHeart, included daily symptom reporting, automated alerts for clinical deterioration, and remote clinician monitoring. The detailed system architecture and workflow are outlined in Multimedia Appendices 1 and 2, respectively. The development of the prototype was also based on the results of previous research using a machine learning method that developed a prediction model for HF readmissions at the same site implemented in a prototype mobile app [15].

### Data Collection

The user acceptance test (UAT) was administered to both health workers and patients. The quantitative study involved posthospitalization patients with an I50 primary diagnosis code (*International Classification of Diseases, 10th Revision*). In the intervention group, participants filled out the Kansas City Cardiomyopathy Questionnaire (KCCQ) and were instructed on the use of the app prototype. A prototype link was given to patients and their families or caregivers, equipped with user-friendly instruments (digital sphygmomanometers, digital body scales, and digital oximeters), and were trained on how to use them. Patients filled out the app prototype daily for 30 days according to the parameters requested and could be monitored online by hospital nurses. If signs required a clinical response, indicated by the presence of green, yellow, and red notifications on the app, patients were followed up on by nurses or cardiologists. Readmission data were obtained from hospital records and verified during follow-up from patient reports. Readmission was defined as all-cause hospitalization within 30 days after index discharge. We used the 23 official and licensed items from the KCCQ from Outcomes Instruments, LLC, which has been translated into Indonesian and tested for reliability and validity, focusing on 4 domains that are closely related to public health: quality of life, self-efficacy, social limitation, and overall summary score [16,17]. Scores were obtained at baseline and 30 days. Missing outcome data were primarily due to incomplete reporting, loss to follow-up, or death during the study period. Adherence was defined as the proportion of days during which patients completed the required daily monitoring entries. After 30 days, participants were asked to re–fill out the KCCQ. In the control group, participants filled out the KCCQ before discharge and 30 days after discharge. No monitoring was carried out at home; only the follow-up schedule at the hospital was followed as usual.

### Data Analysis

Qualitative data were analyzed using thematic analysis [18]. Coding was performed iteratively, and themes were derived through consensus among the researchers. For statistical analysis, baseline characteristics were summarized descriptively. Readmission rates were compared using chi-square tests, and odds ratios (ORs) with 95% CIs were calculated. Changes in KCCQ scores were analyzed using linear mixed-effects models that included all available data without imputation. Due to the pilot nature and limited sample size, multivariable adjustment was not performed to avoid overfitting. A *P* value below .05 was considered statistically significant.

### Ethical Considerations

Ethics approval was granted by the Research and Community Service Ethics Committee, Faculty of Public Health, Universitas Indonesia (Ket-718/UN2.F10.D11/PPM.00.02/2024), and by the institutional review board of the NCVC Harapan Kita (DP.04.03/KEP003/EC001/2024). All participants provided written informed consent.

## Results

### Qualitative Results

A total of 3 cardiologists and 7 nurses participated in the qualitative portion of this study. This qualitative analysis identified 7 major themes reflecting the needs of health care professionals regarding the development of a home-based monitoring system for patients with HF. They can be grouped into 3 overarching domains: digital transformation needs, service delivery optimization, and organizational readiness. The findings indicated that existing care was reactive rather than proactive, fragmented rather than integrated, and manual rather than digital. These limitations contributed to delayed detection of patient deterioration and inefficiencies in care delivery.

### Participant Allocation

Patients who met the eligibility criteria were consecutively recruited during the study period (nonrandomized). A total of 60 patients with HF were enrolled in the study, of whom 30 (50%) were assigned to the intervention group using the FineHeart mobile app, whereas 30 (50%) were assigned to the control group receiving standard care.

During the 30-day follow-up period, 13.3% (4/30) of the patients in the intervention group and 20% (6/30) of the patients in the control group did not complete the quality of life assessment. Therefore, data from 86.7% (26/30) of the patients in the intervention group and 80% (24/30) of the patients in the control group were included in the final KCCQ analysis.

### Baseline Characteristics

The baseline demographic and clinical characteristics of the participants in the quantitative portion of this study are shown in Table 1. Baseline differences were observed between groups in several variables, including educational level and clinical characteristics. Overall, the intervention and control groups showed comparable baseline characteristics in terms of age, gender distribution, and HF diagnosis. However, minor variations were observed in several clinical variables, which should be considered when interpreting the study findings.

**Table 1.** Baseline characteristics.

Variable	Intervention group (n=30)	Control group (n=30)	*P* value
Age (y), mean (SD)	69.0 (4.3)	68.2 (4.1)	.56
Sex (male), n (%)	21 (70)	18 (60)	.42
Marital status (married), n (%)	27 (90)	28 (93.3)	.63
Payer (government health insurance), n (%)	29 (96.7)	30 (100)	.32
Living with partner (yes), n (%)	30 (100)	30 (100)	>.99
Educational level, n (%)			.003
Elementary school	3 (10)	9 (30)	
Junior high school	7 (23.3)	0 (0)	
Senior high school	14 (46.7)	20 (66.7)	
University	6 (20)	1 (3.3)	
Occupation, n (%)			.07
Unemployed	16 (53.3)	24 (80)	
Self-employed	5 (16.7)	1 (3.3)	
Government or private sector employee	9 (30)	5 (16.7)	
Religion, n (%)			.36
Muslim	29 (96.7)	27 (90)	
Catholic	1 (3.3)	1 (3.3)	
Buddhist	0 (0)	2 (6.7)	
Outpatient compliance (regularly), n (%)	23 (76.7)	12 (40)	.004
HF^a^ family history (yes), n (%)	2 (6.7)	4 (13.3)	.39
Smoking (yes), n (%)	7 (23.3)	9 (30)	.56
Alcohol consumption (yes), n (%)	1 (3.3)	2 (6.7)	.55
Substance abuse (yes), n (%)	1 (3.3)	0 (0)	.31
Anemia (yes), n (%)	2 (6.7)	5 (16.7)	.23
Aortic valve disorder (yes), n (%)	3 (10)	0 (0)	.08
Asthma (yes), n (%)	0 (0)	0 (0)	>.99
Cancer (yes), n (%)	0 (0)	0 (0)	>.99
Arrhythmia (yes), n (%)	4 (13.3)	4 (13.3)	>.99
Stroke (yes), n (%)	3 (10)	1 (3.3)	.30
Coronary artery disease (yes), n (%)	12 (40)	7 (23.3)	.17
Dementia (yes), n (%)	1 (3.3)	0 (0)	.31
Diabetes mellitus (yes), n (%)	11 (36.7)	17 (56.7)	.12
Dyslipidemia (yes), n (%)	7 (23.3)	8 (26.7)	.77
Hypertension (yes), n (%)	11 (36.7)	12 (40)	.79
Liver disease (yes), n (%)	0 (0)	0 (0)	>.99
Lung disease (yes), n (%)	6 (20)	2 (6.7)	.13
Protein caloric malnutrition (yes), n (%)	0 (0)	0 (0)	>.99
Psychiatric disorder (yes), n (%)	0 (0)	0 (0)	>.99
Renal disease (yes), n (%)	11 (36.7)	12 (40)	.79
Rheumatic disease (yes), n (%)	4 (13.3)	5 (16.7)	.72
Thyroid disease (yes), n (%)	1 (3.3)	1 (3.3)	>.99
Cardiac devices (yes), n (%)	1 (3.3)	0 (0)	.31
Cardiac surgery (yes), n (%)	1 (3.3)	0 (0)	.31
Coronary angioplasty (yes), n (%)	4 (13.3)	3 (10)	.69
Mechanical ventilator (yes), n (%)	0 (0)	0 (0)	>.99
ACE^b^ inhibitors (yes), n (%)	10 (33.3)	14 (46.7)	.29
ARBs^c^ (yes), n (%)	5 (16.7)	8 (26.7)	.35
ARNIs^d^ (yes), n (%)	11 (36.7)	4 (13.3)	.04
SGLT2^e^ inhibitors (yes), n (%)	1 (3.3)	0 (0)	.31
Diuretics (yes), n (%)	23 (76.7)	29 (96.7)	.02
β-blockers (yes), n (%)	27 (90)	27 (90)	>.99
Spironolactone (yes), n (%)	20 (66.7)	24 (80)	.24
NYHA^f^ classification, n (%)			.49
III	15 (50)	18 (60)	
IV	15 (50)	12 (40)	
LVEF^g^, n (%)			.04
HFrEF^h^	25 (83.3)	17 (56.7)	
HFmEF^i^	1 (3.3)	7 (23.3)	
HFpEF^j^	4 (13.3)	6 (20)	

a HF: heart failure.

b ACE: angiotensin-converting enzyme inhibitor.

c ARB: angiotensin receptor blocker.

d ARNI: angiotensin receptor–neprilysin inhibitor.

e SGLT2: sodium-glucose cotransporter 2.

f NYHA: New York Heart Association.

g LVEF: left ventricular ejection fraction.

h HFrEF: HF with reduced ejection fraction.

i HFmEF: HF with midrange ejection fraction.

j HFpEF: HF with preserved ejection fraction.

### Patient Adherence to the FineHeart App

Among patients assigned to the intervention group, adherence to the FineHeart app varied. Most participants were able to use the app regularly during the monitoring period, although several patients (4/30, 13.3%) demonstrated lower adherence due to difficulties in operating the app or limited caregiver support.

### Readmission Outcome

At 30 days, readmission occurred in 20% (6/30) of the patients in the intervention group, which was lower than the 43.3% (13/30) in the control group. This difference was not statistically significant (*P*=.10), but the OR suggested lower odds of readmission in the intervention group (OR 0.33, 95% CI 0.10‐1.09).

### KCCQ Outcomes



**Figure 1.**
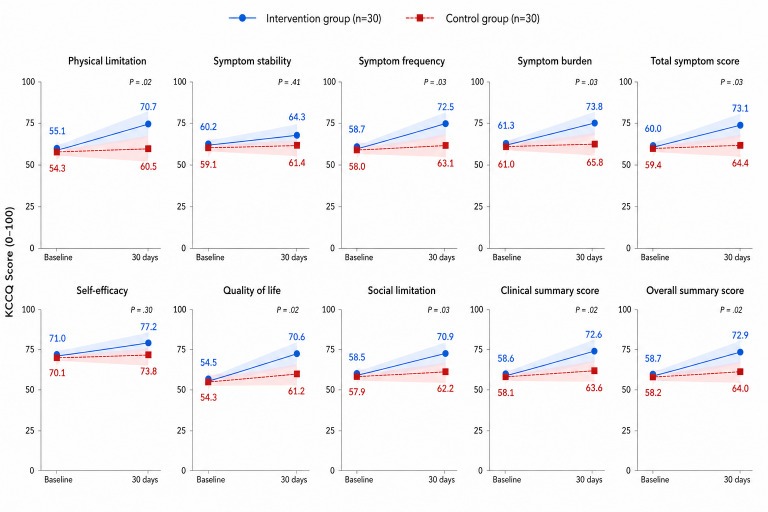
Improvement in Kansas City Cardiomyopathy Questionnaire scores from baseline to 30 days: intervention vs control group. Values shown are estimated marginal means from linear mixed-effects models with fixed effects for time, group, and time × group interaction and a random intercept for participant. Shaded areas represent 95% CIs. Higher scores indicate better health status. *P* values represent the time × group interaction.

Linear mixed-effects analysis demonstrated a significant time-by-group interaction for overall summary score (*P*=.02), indicating greater improvement in the intervention group (Table 2). Improvements were observed in physical limitation, symptom frequency, symptom burden, quality of life, and social limitation. These changes exceeded clinically meaningful thresholds (>5 points). No significant differences were observed for symptom stability and self-efficacy (Figure 1).

**Table 2.** Changes in Kansas City Cardiomyopathy Questionnaire scores (linear mixed-effects model).

Domain	Change in the intervention group	Change in the control group	Interaction *P* value
Physical limitation	+15.6	+6.2	.02
Symptom stability	+4.1	+2.3	.41
Symptom frequency	+13.8	+5.1	.03
Symptom burden	+12.5	+4.8	.03
Total symptom score	+13.1	+5.0	.03
Self-efficacy	+6.2	+3.7	.30
Quality of life	+16.1	+6.9	.02
Social limitation	+12.4	+4.3	.03
Clinical summary	+14.0	+5.5	.02
Overall summary	+14.2	+5.8	.02

### User Acceptance Test

**Figure 2.**
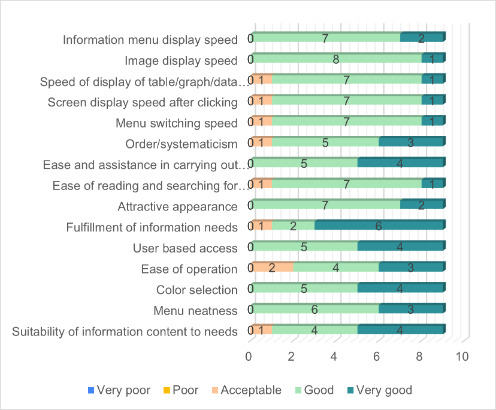
User acceptance test (UAT) results in health workers. Most of the 9 health workers who took part in the UAT provided good and very good ratings in all parameters.

**Figure 3.**
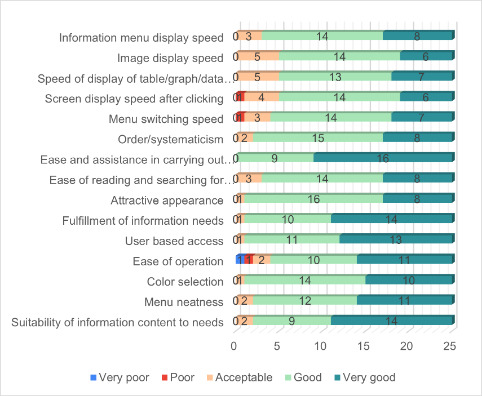
User acceptance test (UAT) results in patients with heart failure. Most of the patients who took part in the UAT (90% on average) provided good and very good ratings in all parameters.

The UAT results among 9 health workers showed that the app prototype was complete and could be used by health care workers and patients at home. The results of the UAT among 25 patients with HF showed that patient acceptance of the FineHeart app prototype was predominantly either good or very good (Figures 2 and 3).

### Missing Data and Mortality

During the 30-day follow-up, some KCCQ data were missing due to incomplete reporting or loss to follow-up. In addition, 3.3% (1/30) of the patients in the intervention group died of non–HF-related causes, and 10% (3/30) of the patients in the control group died due to HF.



## Discussion

### Principal Findings

This pilot study evaluated the feasibility of a digital home monitoring intervention for patients with HF in a resource-limited setting [19]. The findings suggested that the intervention was feasible to be implemented and associated with improvements in patient-reported outcomes, as reflected by significant changes in several KCCQ domains. In addition, a lower proportion of 30-day readmissions was observed in the intervention group than in the control group, although this difference did not reach statistical significance.

Importantly, the improvement in KCCQ overall and domain-specific scores indicated a positive effect on health-related quality of life [20]. These improvements were most pronounced in the physical limitation, symptom frequency, symptom burden, quality of life, and social limitation domains, suggesting that the intervention may support both symptom management and functional status. The observed improvements in KCCQ scores were clinically meaningful as changes exceeding 5 points are generally considered relevant for patients with HF [17]. The magnitude of change observed in this study suggests that patients in the intervention group experienced perceptible benefits in their daily functioning and symptom burden.

In contrast, while the readmission rate was lower in the intervention group (6/30, 20% vs 13/30, 43.3%), the difference was not statistically significant. This might be due to the limited sample size and the pilot nature of this study, which was not powered to detect differences in clinical outcomes.

Taken together, these findings should be interpreted as preliminary signals rather than definitive evidence of effectiveness. The results were hypothesis generating and provided a rationale for larger, adequately powered studies.

An important observation in this study was the difference in mortality between groups [21]. During the follow-up period, 10% (3/30) of the patients in the control group died due to HF, whereas 3.3% (1/30) of the patients in the intervention group died from a non–HF-related cause. This imbalance might reflect differences in baseline clinical stability or disease severity. The higher rate of HF-related mortality in the control group might also partially explain the lower KCCQ scores and higher readmission rates observed in that group. Patients with more advanced diseases or greater instability are more likely to experience worse symptoms, reduced quality of life, and increased health care use.

The findings of this study were broadly consistent with those of previous research on digital and telemonitoring interventions in HF, which have demonstrated variable effects on clinical outcomes but more consistent improvements in patient-reported measures. Several studies have suggested that digital monitoring may enhance early detection of symptom deterioration, improve adherence, and support self-management [13,22]. However, the impact on readmission has been heterogeneous across studies, likely reflecting differences in intervention design, patient populations, health care systems, and implementation strategies [23].

### Study Limitations and Future Research

This study had several limitations that should be considered when interpreting the findings.

First, the study involved a relatively small sample size and was conducted at a single center. As this was a pilot study, the sample size was intended to explore feasibility and preliminary outcomes rather than provide definitive evidence of effectiveness.

Second, this study used a quasi-experimental design without randomization. Although efforts were made to ensure comparability between groups, baseline differences may still have influenced the observed results.

Third, the duration of follow-up was limited to 30 days following hospital discharge. While this period allowed for initial evaluation of patient adherence and early outcomes, longer follow-up periods are necessary to better assess the long-term impact of digital monitoring on hospital readmission and quality of life.

Fourth, not all eligible patients were familiar with smartphone technology, and some declined participation due to concerns about using digital devices. This might limit the generalizability of the findings to populations with lower digital literacy.

Future studies should include larger multicenter cohorts and randomized study designs to better evaluate the clinical effectiveness of mobile-based monitoring systems. In addition, further research should explore strategies to improve patient engagement, the usability of digital platforms, and the integration of remote monitoring systems into routine clinical workflows.

### Conclusions

This pilot study evaluated the feasibility and preliminary outcomes of the FineHeart mobile-based monitoring app for patients with HF following hospital discharge. The findings suggested that the app is feasible for supporting patient self-monitoring and engagement in postdischarge care. Patients who used the app showed favorable trends in several quality of life domains and demonstrated varying levels of adherence to digital monitoring. Although this study was not designed to establish definitive clinical effectiveness, the results provide preliminary signals that mobile-based monitoring may support patient self-management and potentially contribute to improved clinical outcomes. Larger studies are needed to confirm effectiveness.

## Supplementary material

10.2196/84309 Multimedia Appendix 1 FineHeart app system architecture.

10.2196/84309 Multimedia Appendix 2 FineHeart app workflow diagram.

## Abbreviations

HF: heart failure
KCCQ: Kansas City Cardiomyopathy Questionnaire
NCVC: National Cardiovascular Center
OR: odds ratio
UAT: user acceptance test
