# Supplementary figures and images for: Feasibility of a Mobile-Based Home Monitoring System for Patients With Heart Failure: Mixed Methods Pilot Study
Source: JMIR Hum Factors. 2026 Jul 29;13:e84309. doi: 10.2196/84309 (PMC13431228; doi:10.2196/84309)

## SYSTEM ARCHITECTURE: FINEHEART WEB APPLICATION

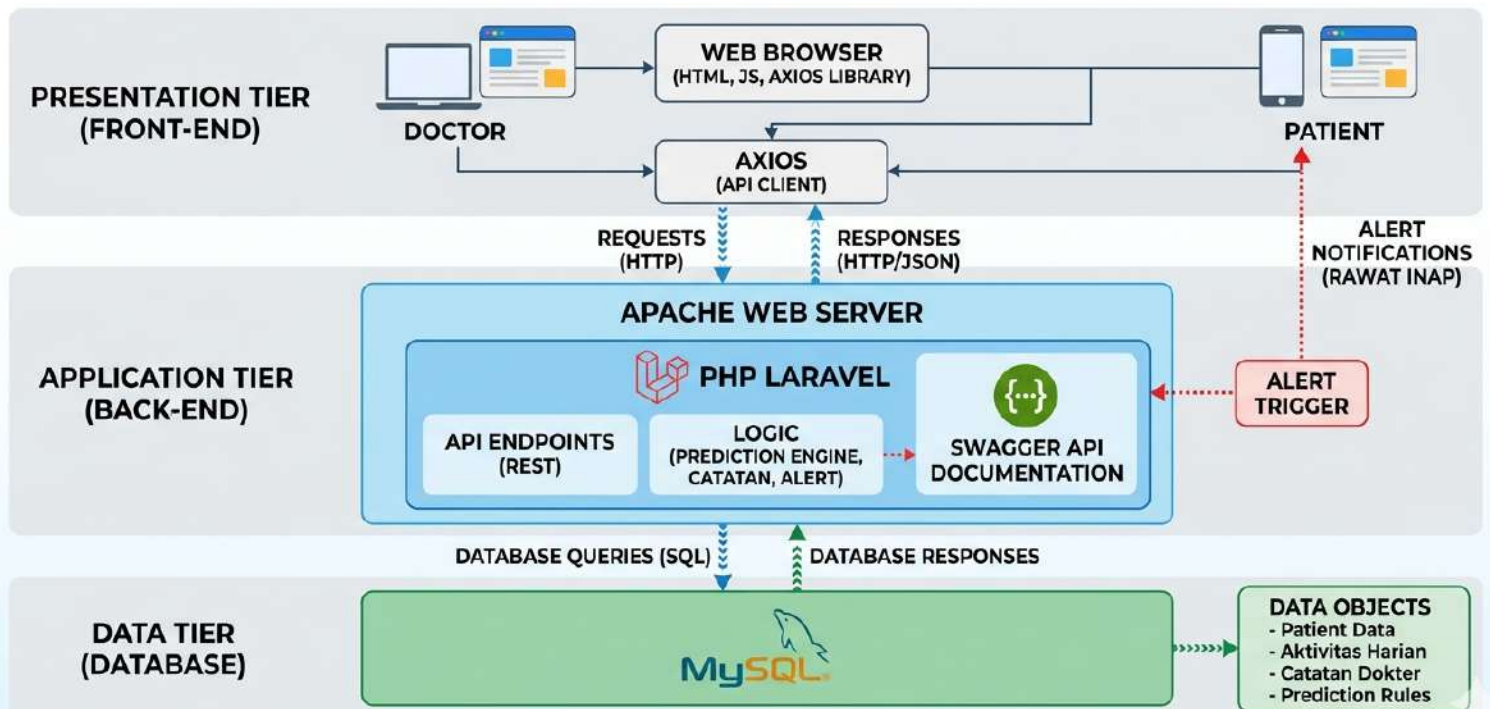

Supplement: Multimedia Appendix 1 [file humanfactors-v13-e84309-s001.pdf]

WORKFLOW DIAGRAM: FINEHEART PATIENT MONITORING AND ALERT SYSTEM

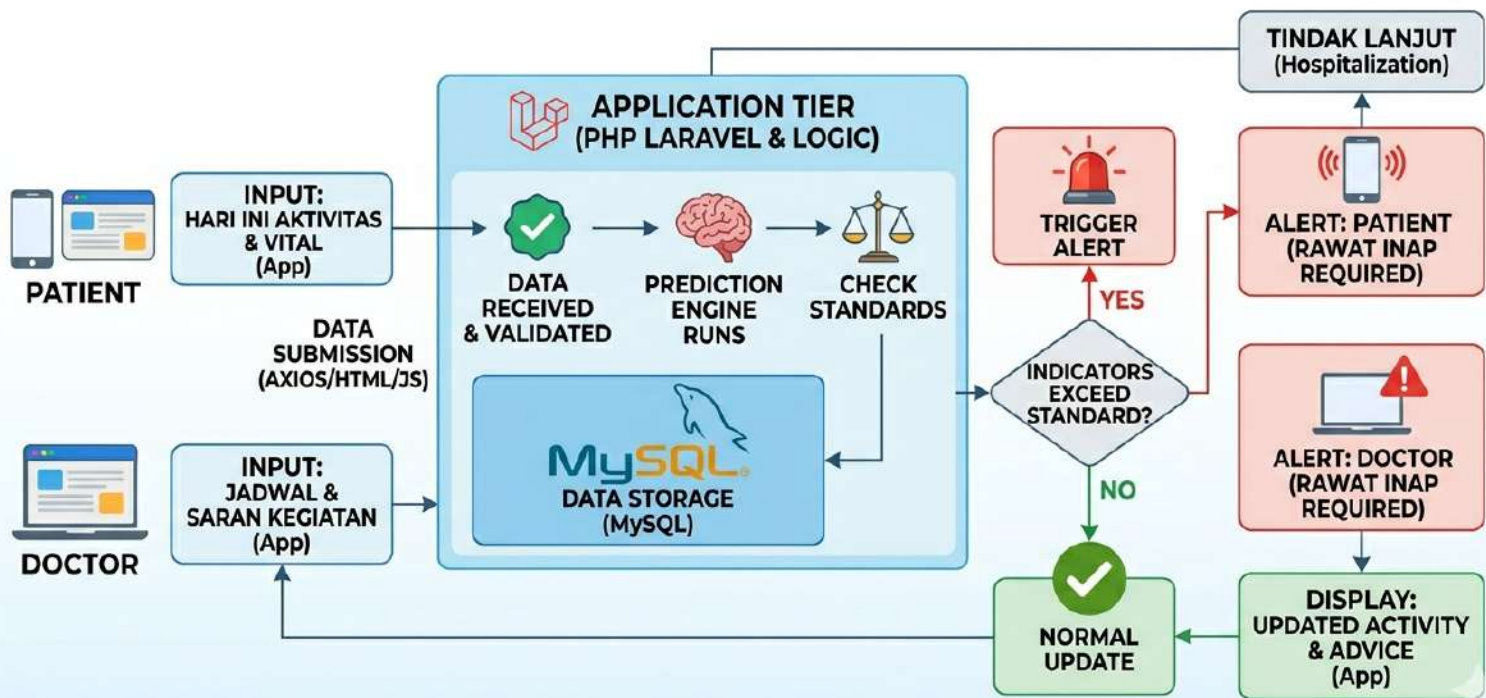

Supplement: Multimedia Appendix 2 [file humanfactors-v13-e84309-s002.pdf]
